# Supplementary material for: Mini-TAS as a confirmatory mapping tool for remapping areas with uncertain filarial endemicity to exclude/ include for mass drug administration: A report from field validation in India
Source: PLoS One. 2023 Nov 3;18(11):e0293641. doi: 10.1371/journal.pone.0293641 (PMC10624291; doi:10.1371/journal.pone.0293641)
Supplement: Attachment — Submitted filename: Panda et al_ONE-D-23-20937_comments.docx [file pone.0293641.s001.docx]

**Review comments: Manuscript PONE-D-23-20937**

**General comment**

After reading the entire manuscript I think it is useful to make it more clear to the reader about the design of the study. In my opinion the study simply employed a cross-sectional design with two sampling strategies – purposive and random cluster sampling to evaluate the performance of four (4) sampling methods for LF remapping mapping in India. The four surveys methods are: mini-TAS, community-based household surveys of individuals aged from 5 years (national remapping protocol), community-based household surveys of individuals aged from 10 years in villages/wards with schools for mini-TAS, and entomology based molecular xenomonitoring. This was done with the aim of identifying the most useful survey method to identify districts that might have ongoing LF transmission in India and thus be considered for MDA. If the authors agree, the title may then need to be aligned with the broad objective of the study.

**Specific comments**

1. The abstract needs to be revised for clarity. Reading the methodology and results, one notes that there were four (4) survey methods being compared as mentioned in the general comment above.
2. Abstract, line 32: In the methodology, line 151, the Mf survey under the mini-TAS areas was not actually among adults only, but community members 10 years old and above.
3. Abstract, line 30-33: The statement that reads, ‘Purposive sampling … sampling’ is not clear.
4. Abstract, line 33-36: The statement that reads, ‘Costing of … Mini-TAS’ is not clear.
5. Introduction, line 76: The references cited (6 and 7) do not include any WHO guidance, but rather operational research studies in Ethiopia and Tanzania in sub-Sahara Africa.
6. Methodology, Study area, lines 94 and 97: Check the position of commas among the digits in the population numbers.
7. Methodology, Sampling strategies and Survey plans, line 106: Replace the word ‘uses’ with ‘used’.
8. Methodology, Purposive sampling, line 116: In the introduction, line 72, it is stated that eight (8) sites are purposively sampled in the national remapping protocol. Why were five (5) sites sampled for this study?
9. Methodology, Purposive sampling, line 120: Replace the word ‘aging’ with ‘age’.
10. Methodology, Purposive sampling, line 122: Revise the word ‘sapling’ to ‘sampling’.
11. Methodology, Mini-TAS, line 137: The reference cited is not appropriate for Mini-TAS – it is for an LF entomology handbook.
12. Methodology, Mini-TAS, line 143-145: This statement reading ‘In sites … sampling’ is not clear. The sampling for mini-TAS should be done as per the Confirmatory Mapping Survey Sample Builder (SSB) tool (<https://www.cor-ntd.org/resources/confirmatory-mapping-survey-builder>). It is important to cite this resource if it was used.
13. Methodology, Molecular xenomonitoring, line171: Delete the word ‘by’.
14. Methodology, Molecular xenomonitoring, line173: Add the word ‘which’ after the close parenthesis.
15. Methodology, Filarial DNA extraction from mosquito pools, line188: Write the proper chemical name of magnesium chloride (MgCl_2_).
16. Methodology, Data analysis, line 214-215: Correct the species name to ‘*W. bancrofti*’.
17. Ethical statement, line 220: Use past tense to read, ‘The study was …’
18. Results, Purposive sampling, Kalahandi district, line 246: Please provide a brief description about the cadre of staff referred to as ASHAs.
19. Results, Table 1: Please provide more information about the 3 data sources of chronic LF cases in the districts (NVBDCP, ASHAs, and Research).
20. Results, Tables 3, 4, 5, 7,8: Please consider presenting the results in simple tables and/or figures.
21. Results, Purposive sampling, Kalahandi district, line 258: replace the word ‘is’ with ‘as’.
22. Results, line 284-285: There seems to be a missing word(s) in the statement reading, ‘As many ... females.
23. Discussion, line 388-390: Please check grammar in the sentence.
24. Discussion, line 392-394: Check accuracy of statement.
